# Characterization of *Clostridioides difficile* Strains, the Disease Severity, and the Microbial Changes They Induce

**DOI:** 10.3390/jcm9124099

**Published:** 2020-12-18

**Authors:** Hanan Rohana, Maya Azrad, Orna Nitzan, Amos Adler, Dana Binyamin, Omry Koren, Avi Peretz

**Affiliations:** 1 The Azrieli Faculty of Medicine, Bar Ilan University, Safed 1311502, Israel; hanan.rohana@gmail.com (H.R.); ONitzan@pmc.gov.il (O.N.); dsimoni925@gmail.com (D.B.); Omry.Koren@biu.ac.il (O.K.); 2 Baruch Padeh Medical Center, Clinical Microbiology Laboratory, Poriya, Tiberias 1528001, Israel; mazrad@poria.health.gov.il; 3 Baruch Padeh Medical Center, Unit of Infectious Diseases, Poriya, Tiberias 1528001, Israel; 4 Tel Aviv Sourasky Medical Centre, Microbiology Laboratory, Tel Aviv 6423906, Israel; amosa@tlvmc.gov.il; 5 The Sackler Faculty of Medicine, Tel Aviv University, Tel Aviv 69978, Israel

**Keywords:** *C. difficile*, MLST, clade, disease severity, microbiome

## Abstract

Background: *Clostridioides difficile* infection (CDI) is a major nosocomial disease. The characteristics of different strains, the disease severity they cause, their susceptibility to antibiotics, and the changes they inflict on gut microbiome, have not been comprehensively studied in Israel. Methods: A severity score was calculated for 70 patients. Stool samples were tested for toxins presence using a special kit. Bacteria were isolated, identified by matrix-assisted laser desorption ionization-time of flight (MALDI-TOF) and antibiotic susceptibility tests were performed for several antibiotics. Strains were classified by Multi-locus sequence typing (MLST), and changes in gut microbiome were tested. Results: ST04 (22.5%) and ST37 (12.7%) were the most frequent strains. Clade (phylogenetic lineage) 1 was the most (81.4%) prevalent. We found significant associations between ST and age (*p* = 0.024) and between ST and moxifloxacin susceptibility (*p* = 0.001). At the clade level, we found significant associations with binary toxin gene occurrence (*p* = 0.002), and with susceptibility to both metronidazole and vancomycin (*p* = 0.024, 0.035, respectively). Differences in intestine microbiome were affected by age, clades’ distribution and STs. Conclusions: By defining the characteristics of the different strains and clades, clinicians can choose medical interventions based on the predicted response or disease severity associated with each strain, enabling new advances in the field of personalized medicine.

## 1. Introduction

In recent years, *Clostridioides difficile* infection (CDI) has become a major nosocomial disease, associated with serious morbidity and potential mortality [1]. Symptoms include diarrhea, fever, abdominal pain, and leukocytosis. A severe infection might lead to pseudomembranous colitis and toxic megacolon, which, in extreme cases, can be lethal. The risk factors for CDI include age over 65, hospitalization in an intensive care unit (ICU), background of inflammatory bowel diseases, chemotherapy, and antibiotic therapy [2]. As antibiotics provide a convenient niche for spore germination, and contribute to CDI virulence [3], they are the main risk factor for this infection, with symptoms usually appearing among patients who received various combinations of microbiota-disrupting antibiotics.

The association between CDI and prior use of antibiotics led to the understanding that disturbances in the gut microbiota, i.e., the collection of symbiotic microorganisms that live inside and on the human body, is likely to play an essential role in CDI. Imbalances in these microbial communities, also known as dysbiosis, may lead to disrupted host-microbe interactions. This disturbance occurs mainly following antibiotic administration. Extended findings suggest that such dysbiosis results in an increased risk of *C. difficile*-associated diarrhea [4,5]. Little is known regarding the difference in the microbiome structure of patients who were treated with different types of antibiotics before the emergence of CDI. Furthermore, the effect of different *C. difficile* strains on the gut microbiome is unknown to date.

Most *C. difficile* strains produce toxins, toxin A and toxin B [6]. Some strains secrete a third toxin—the binary toxin. For example, the hyper-virulent ribotype 027/NAP1, which has been responsible for several outbreaks of CDI in health care facilities [7]. NAP1 is highly resistant to fluoroquinolone antibiotics, produces higher amounts of toxins in comparison with other strains. In Israel, several outbreaks caused by this strain were identified in long-term care facilities and in general hospitals [8].

Despite the centrality of strain identification, particularly for epidemiological surveillance and evidence-informed decision making in public health, such analysis is not routinely performed. CDI diagnosis is usually determined using molecular biology methods based on identification of the toxin genes [9], since culturing of *C. difficile* last for 48 h and most healthcare facilities prefer to identify and isolate the patients more quickly. Furthermore, strains identification by typing or sequencing methods is expensive and requires specific equipment that is not available in all laboratories. As a result, only limited data are available regarding strains that are associated with a severe disease compared to those that cause mild diarrhea or asymptomatic colonization. Furthermore, the characteristics of different strains, the severity of the disease they cause, and their susceptibility to antimicrobial agents, have not yet been thoroughly studied in Israel. This study set out to explore the epidemiology and the clinical characteristics of CDI by classifying the different strains using the Multilocus Sequencing Typing (MLST) method. Additionally, we tested whether different strains cause diverse changes in the human microbiome. The findings will help consolidate treatment strategies, especially in hospitalized patients.

## 2. Experimental Section

### 2.1. Study Population

The study group included 70 patients aged ≥18 years, who suffered from diarrhea, were hospitalized at the Baruch Padeh Medical Center Poriya, Israel, between January 2016 and April 2018, and were diagnosed with CDI via samples routinely collected and tested at the Clinical Microbiology Laboratory. Overall, 94 CDI patients were identified at the study period; however, 24 were excluded due to inflammatory bowel disease, history of CDI, gastrointestinal bleeding, or laxative use. There were no cases of recurrences.

The study was approved by the Ethics (Helsinki) Committee of the hospital (Approval no. POR-0085-15) and of superior Helsinki board of the Israeli health ministry. Every subject signed a consent form before enrollment.

All CDI cases were confirmed by stool examination for toxigenic *C. difficile*, identifying three targets: toxin B, binary toxin, and *tcd*C deletion using the GeneXpert *C. difficile* PCR assay (Cepheid, Sunnyvale, CA, USA).

### 2.2. Bacterial Isolation and Identification

All stool samples were inoculated on a selective growth medium—chromID™ *C. difficile* (CDIF) (bioMérieux, Durham, NC)—and then incubated at 37 °C, under anaerobic conditions, for 48 h. *C. difficile* colonies appear asymmetric and black-colored. Final identification was performed by matrix-assisted laser desorption ionization-time of flight (MALDI-TOF) mass spectrometry, using the Bruker Biotyper system (Bruker Daltonics, Bremen, Germany) [10]. All isolates were kept in beads in −80 °C until further use.

### 2.3. Multi-Locus Sequence Typing

Total genomic DNA was extracted from all bacterial isolates using the QIAamp DNA Mini Kit (QIAGEN, Hilden, Germany), according to the manufacturer’s instructions. MLST was performed in two stages, as previously described [11].

#### 2.3.1. Amplification (A)

PCR amplicons for 7 housekeeping genes were obtained for each isolate as previously described [11], using the qPCRBIO SyGreen Blue Mix Hi-ROX kit (PCR Biosystems Inc., Wayne, PA, USA), on a real-time polymerase chain reaction (PCR) device (BioRad CFX96 Real-Time Detection System, Hercules, CA, USA). Amplification conditions were: 95 °C for 2 min to ensure polymerase activation, followed by 40 cycles of (95 °C for 5 s, and 60–65 °C for 20–30 s). The amplification products were characterized by melting curve analysis subsequent to the amplification run.

#### 2.3.2. Sequencing and Sequence Type (ST) Determination (B)

Prior to sequencing, the PCR products were purified and their nucleotide sequences on each DNA strand were determined using amplification primers [11].

Genotyping was performed with an ABI PRISM^®^ 310 Genetic Analyzer (Applied Biosystems, Foster City, CA, USA), using the capillary electrophoresis method. The reaction mix (per sample) consisted of 1 μL PCR product, 12 μL Hi-Di formamide, and 0.5 μL GeneScan™ 500 ROX™ size standard (Thermo Fisher Scientific, Waltham, MA, USA). The data were analyzed using ChromasLite v2.01 (Technelysium DNA Sequencing Software, South Brisbane, Australia) and Sequencher v5.1 (Gene Codes Corporation, Ann Arbor, MI, USA).

Following sequencing of the 7 housekeeping genes found in each specific strain, the allelic numbers of each gene and the STs were assigned using the PubMLST *C*. *difficile* database (http://pubmlst.org/cdifficile/). The ST number was assigned for each specific combination of alleles. Using phylogenetic analysis, the sequences of the genes were used to assess the evolutionary relationship of the different strains, enabling the identification of clades, i.e., phylogenetic lineages.

### 2.4. Toxin Detection

#### 2.4.1. Toxin Gene Detection by PCR (A)

A multiplex real-time PCR assay was designed to target and detect the *cdtA* (binary toxin), *tcdA* and *tcdB* (toxin A and B, respectively) genes in a single PCR run using specific primers [12]. For this purpose, 1 µL of the DNA sample tubes containing 24 µL reaction mixture (0.8 µM of each primer, 0.4 µM of each fluorophore probe, 6 mM MgCl_2_, 200 µM dNTPs, 1 IU Super-Hot Taq polymerase, and 1 × PCR buffer). The assay was run on the Bio-Rad CFX96 Real-Time System, as previously described. The cycler conditions were denaturation of the pre-amplified templates at 95 °C for 15 min, followed by 45 cycles of denaturation at 95 °C for 15 s, and annealing and extension at 60 °C for 60 s [12].

#### 2.4.2. Toxin Detection Using a Chromatographic Immunoassay (B)

The presence of *C. difficile* toxins in fecal samples was determined using the CerTest *Clostridium difficile* GDH + Toxin A + B combo card test kit (Certest Biotec, S.L, Zaragoza, Spain), according to the manufacturer’s protocol. This is a chromatographic immunoassay for the simultaneous qualitative detection of *Clostridium difficile* antigens toxin A, toxin B, and glutamate dehydrogenase (GDH), an enzyme produced in large quantities by all strains [13].

### 2.5. Antibiotic Susceptibility Testing

Antimicrobial susceptibility testing (AST) was performed using the Etest quantitative technique, in order to determine the minimum inhibitory concentration (MIC) values, i.e., the minimal concentration (μg/mL) of a given antibiotic that inhibits the growth of a particular bacterium under specific experimental conditions. Once isolated, *C. difficile* colonies were suspended in thioglycollate broth medium (Becton Dickinson, Heidelberg, Germany) until 1.0 McFarland turbidity. Using a sterile cotton swab, the inoculum was spread on Brucella blood agar growth medium (Hy Laboratories, Rehovot, Israel). A gradient Etest strip (bioMérieux, Durham, NC) of the following antibiotics was added to the plates: vancomycin, metronidazole, moxifloxacin, and tigecycline. The plates were incubated under anaerobic conditions, at 37 °C, for 24 h. MIC was determined for each antibiotic. The interpretation of test results (sensitive/resistant) was in accordance with the European Committee on Antimicrobial Susceptibility Testing (EUCAST) recommendations, that determined epidemiological cut-offs (ECOFFs) [14].

### 2.6. Disease Severity Scoring and Demographic Data Collection

Disease severity was scored using the severity score index (SSI), developed by Velazquez-Gomez et al. [15]. This method, each of the following criteria are assigned one point: altered mental status, abdominal pain or distention, tachycardia (≥110 beats/min), hypotension (mean arterial pressure < 65 mm Hg), fever (≥38.3 °C), leukocytosis (WBC > 20,000) or leucopenia (WBC < 1500), or increased band forms (>10% bands); ascites or colitis documented by imaging studies; hypoalbuminemia (<2.5 mg/dL); admission or transfer to an intensive care unit. CDI with an SSI of 1–3 is defined as a mild disease, 4–6 is considered moderate, and ≥7 is considered severe.

The following demographic data were retrospectively collected from patient medical records: gender, age, community versus nosocomial-acquired CDI (community = development of CDI within the 48 h of hospitalization, 30-day mortality due to CDI, and number of antibiotics classes (different antibiotic families) taken.

### 2.7. Gut Microbiome

#### 2.7.1. DNA Extraction (A)

DNA was extracted from stool samples using the Power Soil DNA Isolation Kit (MoBio, Carlsbad, CA, USA), according to the manufacturer’s instruction, with an initial step of bead-beating for 2 min.

#### 2.7.2. DNA Amplification (B)

The variableV4 region of the bacterial 16 S rRNA gene was amplified by PCR, using the 515 F and 806 R barcoded primers, according to the Earth Microbiome Project protocol [16].

A unique barcode sequence was applied to each sample to distinguish between them. For each PCR tube, the following materials were added: 2 μL 806R (reverse, 10 μM) primer, 2 μL 515 F (forward, 10 μM) primer, 17 μL ultra-pure water, 25 μL Primestar max PCR mix (Takara Bio USA Inc., Mountain View, CA, USA), and 4 μL DNA. The reaction conditions were: 30 cycles of denaturation (98 °C for 10 s), annealing (55 °C for 5 s), and extension (72 °C for 20 s), with final elongation at 72 °C (for 1 min).

#### 2.7.3. DNA Purification (C)

The PCR products were purified using AMPure magnetic beads (Beckman Coulter, Brea, CA, USA), according to the manufacturer’s protocol [17].

#### 2.7.4. DNA Quantification and Pooling (D)

Purified DNA was quantified using the Qubit dsDNA HS assay (Thermofisher, Waltham, MA, USA), according to the manufacturer’s protocol [18]. Following the measurement, samples were pooled at equal concentrations (50 ng/μL).

#### 2.7.5. Pooling Purification (E)

Prior to sequencing, the DNA was further purified; 2% E-Gel agarose was inserted in an E-Gel PowerBase device. DNA fragments were cut and purified from the gel, using NucleoSpin^®^ Gel and PCR Clean-up kit (Macherey-Nagel, Duren, Germany), according to the manufacturer’s protocol.

#### 2.7.6. Sequencing (F)

Purified DNA products were sequenced using the Illumina MiSeq platform, at the Genomic Center, Azrieli Faculty of Medicine, BIU, Israel.

#### 2.7.7. Analysis (G)

Data analysis was performed using QIIME2 [19]. To this end, paired-end sequences were joined, sequence reads were multiplexed by per-sample barcodes, and Illumina-sequenced amplicon read errors were corrected using the Divisive Amplicon Denoising Algorithm (DADA2) [20]. In addition, a phylogenetic tree was generated. Alpha and beta diversity calculations, describing diversity within and between samples, respectively, were performed using a feature table on samples containing at least 8,000 sequences. For beta diversity, principal coordinate analysis (PCoA) was performed using both weighted and unweighted UniFrac distances [21]. Evenness and richness, two alpha diversity parameters, were calculated using Faith’s Phylogenetic Diversity and Pielou’s Evenness measures [22,23].

In addition, LEfSe was performed [24], to identify the features that are significantly different between samples according to relative abundances.

### 2.8. Statistical Analysis

Chi-squared or Non-parametric Wilcoxon–Mann–Whitney Rank Sum Test for independent samples were applied for analyzing the differences in distribution of categorical or continuous parameters, respectively, between the different strains. The tests applied were two-tailed, and a *p*-value of 5% or less was considered statistically significant. Composite descriptions of amplicon sequence variants (ASVs) were obtained using PCA and ICA. The association of these composites was checked with the discrete (ANOVA) and the continuous (Spearman correlation) variables, as well as Factor Analysis of Mixed Data.

## 3. Results

### 3.1. Demographic and Microbial Characteristics

Seventy CDI patients were enrolled in this study. The average age of the study population was 74.6 ± 11.5 years, and there were similar percentages of females and males (Table 1). Within the study group, 47 (67.1%) CDI cases were nosocomial, compared with 23 (32.9%) cases that were acquired in the community. As for disease severity, 45 (64.3%) patients had mild disease, 23 (32.8%) patients had moderate disease, and two (2.9%) patients had severe disease. The 30-day mortality rate was 31.4%.

Three isolates (4.3%) were typed as NAP1/027/B1. All strains were divided according to the different toxins they produced (according to the CerTest *Clostridium difficile* GDH + Toxin A + B combo card test kit); eight (11.4%) strains produced toxin A, 23 (32.9%) strains produced toxin B, and 39 (55.7%) produced both toxins. All strains had the genes for both toxins A and B. Furthermore, nine (12.9%) isolates had the binary gene, while 61 (57.1%) lacked it.

### 3.2. Antibiotic Susceptibility Testing

Out of the 70 isolates, 5 (7.1%) were found resistant to tigecycline; the mean MIC_90_ was 0.5 µg/mL. Twelve (17.1%) isolates were resistant to metronidazole; the mean MIC_90_ was 40.6 µg/mL. In addition, 29 (41.4%) of the isolates were resistant to moxifloxacin; the mean MIC_90_ was 13.7 µg/mL. Finally, one isolate (1.4%) was resistant to vancomycin, with a MIC_90_ of 4.2 µg/mL. Results are presented in Table 2.

### 3.3. Associations Between STs, Epidemiological Data, and Bacterial Characteristics

**Figure 1:**
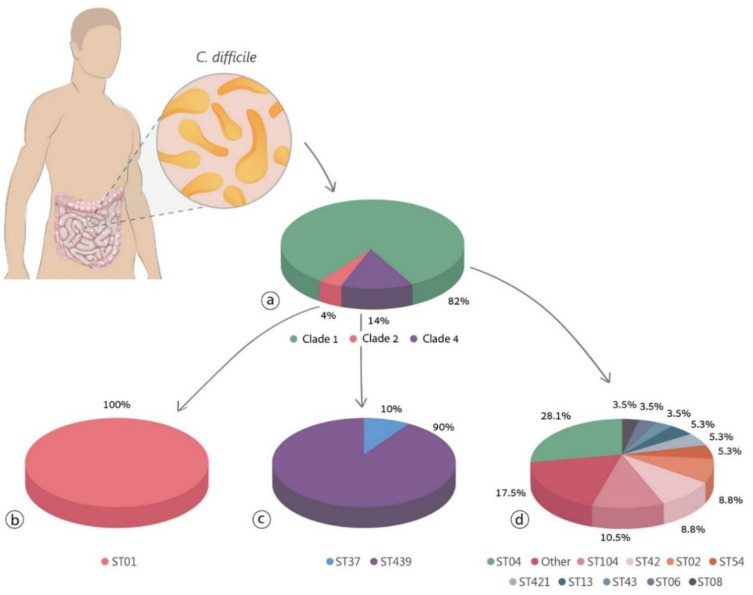
The prevalence of clades and sequence typing (STs) in the study. (**a**) Prevalence of the different clades. (**b**) The prevalence of the different STs in clade 2. (**c**) The prevalence of the different STs in clade 4. (**d**) The prevalence of the different STs in the study.

STs analysis found 16 isolates (22.5%) ST04, 9 (12.7%) ST37, 6 (8.5%) ST104, 5 (7%) ST42, and 5 (7%) ST02. The prevalence of all other STs was lower than 5%. In all, 57 isolates (81.4%) belonged to clade 1, 10 (14.3%) belonged to clade 4, and 3 (4.3%) belonged to clade 2 (Figure 1).

Due to the fact that some STs were present in fewer than three isolates, subsequent analysis focused on the five most frequent STs: ST04, ST37, ST104, ST42, and ST02. All other STs were grouped and defined as “other”. The disease severity was not significantly different between the different ST groups (*p* = 0.105) (Table 3). Furthermore, no significant associations were found between ST and other epidemiological data.

Similarly, none of the bacterial characteristics associated with ST (Table 4). No statistically significant association was found between ST and susceptibility to metronidazole (*p* = 0.55); all ST104 isolates (*n* = 6), and ST42 isolates (*n* = 5), as well as 80% of ST02 isolates, were sensitive to metronidazole. This was also true for vancomycin (*p* = 0.230), and tigecycline (*p* = 0.626). In contrast, a statistically significant association between susceptibility to moxifloxacin and ST (*p* = 0.001) was seen; 15 ST04 isolates (93.7%) were resistant, while all ST42 (*n* = 5) and ST104 isolates (*n* = 6) were sensitive.

In contrast, a significant association was observed between ST and age (*p* = 0.024). The lowest mean age was of patients with ST104 (*n* = 6), 61.67 ± 18.8 years; the highest mean age was of patients with ST37 (*n* = 9), 79.67 ± 10.6 years.

### 3.4. Associations Between Binary Toxin Gene Occurrence, Epidemiological Data, and Bacterial Characteristics

The binary gene was detected in 9 isolates, while 61 lacked it. There were no significant differences in the epidemiological characteristics of the binary-positive versus -negative groups (see Table S1).

All three NAP1 isolates belonged to the binary-positive group (*p* = 0.001). No significant differences in the production of toxins A and B were found (*p* = 0.449). However, all isolates that produced toxin A only (*n* = 8, 13.1%) belonged to the non-binary group. There were no significant differences between the two groups in terms of infection acquisition (*p* = 0.895), number of antibiotic classes taken prior to CDI diagnosis (*p* = 0.454), or 30-day mortality rates (*p* = 0.368). A significant association was found between binary toxin gene presence and susceptibility to metronidazole (*p* = 0.02) and vancomycin (*p* = 0.009). In the binary-negative group, 53 isolates (86.9%) were sensitive to metronidazole while 8 (13.1%) were resistant. Moreover, in the binary-negative group, all 61 isolates (100%) were sensitive to Vancomycin. The results are summarized in Table 5.

### 3.5. Microbiome Analysis

**Figure 2:**
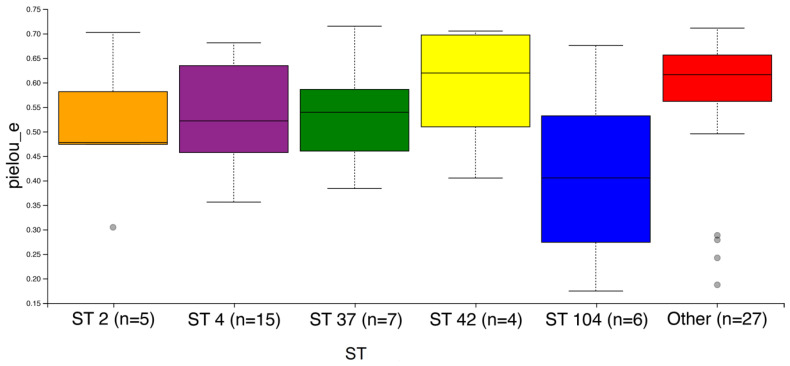
Pielou’s Evenness plot, a measure of community evenness among the different STs.

Fecal microbiota alpha and beta diversity analyses found no significant difference between the different ST groups of isolates. However, evenness calculated using Pielou’s test showed a significant association with ST, with lower Pielou values calculated in the ST104 group (*p* = 0.04) as compared to the less frequent STs (other) (Figure 2). Similar analyses by isolate clade found no significant difference between the groups. The LEFSE analysis found *Veillonella* spp. to significantly higher in clade 1, compared to *Prevotella*, *Bacteroides*, *Mogibacterium*, and other species, which were significantly higher in clade 4.

## 4. Discussion

The current study describes the application of a molecular typing method, MLST, for the characterization of different *Clostridioides difficile* strains. The major aim was to understand the role of strain differences in the severity of CDI.

### 4.1. Characteristics of the Study Population

The present study group included 70 patients of an average age of 74.6 ± 11.5 years, which emphasizes that CDI is a disease of the elderly (>65 years old), with both incidence and severity markedly increasing in this population [25]. In the studied cohort, 47 CDI cases were nosocomial (67.1%), which is consistent with the classic assumption that *C. difficile* infection is a hospital-acquired infection.

Twenty-two patients (31.4%) died due to CDI, consistent with the reported mortality rate, which ranges from 6% to 30% [26]. However, CDI is often comorbid with other diseases, and thus might not be the major cause of death in some cases. Disease severity was mild in 63.3% of the patients, moderate in 32.8% of the patients, and severe in 2.9% of the patients. This distribution is consistent with a recent study in northern Israel reporting that 70.4% of CDI cases were mild, and 29.6% were moderate [27].

### 4.2. The Characteristics of the Different STs

No significant associations between ST and gender, toxin production, disease severity, type of infection acquisition (nosocomial/community-acquired), 30-day mortality, or binary toxin expression were found. However, a significant association was found between ST and patient age. ST104 was more prevalent among younger patients compared to ST04 and ST37, which were more prevalent in older patients. This might be due to the facts that both STs 37 and 04 were the most frequent STs, that CDI is associated with older age, and that the average age of the study population was 74.6 years. In several international studies, ST02 showed a 6.3% world-wide frequency, ST42 showed 4.6%, and ST37 showed 1.2% [28], statistics which do not match the present distribution, where 12.7% were ST37, 7% were ST42, and 7% were ST02. These findings suggest that ST distribution differs regionally across the world. The prevalence of strain ST37 found here matches its reported prevalence in two tertiary-care hospitals in Shandong Province, China, where it constituted 12.5% of the total isolates [29]. In that study, the strain was *tcdA*-negative, *tcdB*-positive, and *cdtA*/*cdtB*-negative (A − B + CDT−). In contrast, the ST37 isolate in the present study all isolates were *tcdA*-positive produced toxin, and five isolates produced both toxins A + B. Furthermore, out of nine isolates that possessed the binary gene, two (22.2%, 2/9) belonged to ST37, i.e., ST37 could have a profile of A + B + CDT+. These differences are possibly attributable to recombination effects.

With respect to antibiotic susceptibility, a significant association was found between ST and susceptibility to moxifloxacin, a fluoroquinolone antibiotic: 93.7% of ST04 isolates were resistant, compared to ST104 and ST42, which were all sensitive (100%). The three NAP1 cases in this study (ST1) were all resistant to moxifloxacin. It should be noted that this antibiotic is not generally used for the treatment of CDI. However, a high resistance rate to this antibiotic could predict an infection with a virulent strain or a therapeutic failure [30]. Regarding the other antibiotics, most STs showed high sensitivity to metronidazole (82.9%), to vancomycin (98.6%), and to tigecycline (92.9%). Metronidazole and vancomycin are considered first-line treatments for CDI. A survey of the molecular epidemiology of *C. difficile* in Israel showed that 18.3% of the strains were resistant to metronidazole [8]. This matches the present results, given that 17.14% of the strains were resistant. On the other hand, the rate of vancomycin-resistant isolates in the same survey, which included 57 NAP1 isolates, was 47% [8]. The present study found a 1.4% resistance rate to vancomycin. This difference may indicate on different frequency of vancomycin treatment for *C. difficile* in the different hospitals. It is also possible that the previous study included isolates that were part of an epidemic and therefore were all vancomycin-resistant.

### 4.3. The Prevalences of the Different Clades

The current study showed that 81.4% of the isolates belonged to clade 1, 14.3% belonged to clade 4, and 4.3% belonged to clade 2 (NAP1). When comparing these results to a study conducted in 2017, of the frequency of STs and clades reported across the world [28], clade 1 was the most abundant, with a frequency of 57.7%, followed by clade 2 with a prevalence of 29.1%, and clade 4 with a prevalence of 3.4%. Clade 1 was the most frequent in most countries, but were characterized as negative for binary toxin, and positive for toxins A and B. This is in contrast to the current population, in which 7% of the STs belonging to clade 1 were positive for the binary gene.

### 4.4. The Binary Gene and Susceptibility to Antimicrobial Agents

The binary gene was absent in 61 isolates (87.1%). A significant association was found between gene presence and sensitivity to vancomycin; all strains that lacked the binary gene were sensitive to vancomycin, while 88.9% of the strains that had the binary gene were sensitive. The resistant isolate belonged to ST37, and was resistant to all four antibiotics tested in this study. In a similar study conducted in Florida in 2017, 82.6% of the A + B + CDT+ isolates were sensitive to vancomycin, with 17.4% demonstrating intermediate resistance [31]. As for metronidazole, almost half of the binary gene-positive isolates in the present study were resistant to the antibiotic, while 86.9% of those lacking the gene were sensitive. When looking again at the study conducted in Florida, all the isolates that were positive for the binary gene, were sensitive to metronidazole [31]. The binary toxin has been associated with the increased virulence and spread of *C. difficile,* especially NAP1 [32]. However, 55.5% of the binary gene-positive isolates in the current study caused mild disease, compared to 44.5% that caused moderate disease. Thus, the presence of the binary gene does not necessarily enhance the severity of CDI, but did show an association with pathogen resistance to metronidazole and to vancomycin.

### 4.5. Characterization of the Intestinal Microbiota of CDI Patients

According to the taxonomic distribution, no clear differences in microbiome composition were detected in patients infected with the different STs, nor the different clades, although some distinctions were noted. For example, the calculated Pielou values, a measure of community evenness, were lower in the ST104 group (*p* < 0.05) compared to the less frequent STs (other), indicating reduced bacterial diversity within this particular group. The mean age of the ST104-positive patients was the lowest compared to the other groups. Such data contradict the hypothesis that older individuals are expected to display a loss of diversity-associated taxa compared to younger ones [33]. The discrepancy between studies may be related to diet and the misuse of antibiotics, necessitating a deeper investigation of their impact on the microbiome across different populations.

On the clade level, the LEFSE analysis identified a significant enrichment for *Veillonella* in clade 1, compared to *Prevotella*, *Bacteroides*, and others, which were significantly enriched in clade 4. In general, *Veillonella* is enriched in CDI patients, and increased in PPI users [34,35]. The high abundance of *Veillonella* in this particular group is likely related to massive vancomycin treatment [36], which is usually applied in severe cases [37].

Previous studies comparing the fecal microbiota composition in healthy older subjects, young adults, children, and older patients diagnosed with CDI [38,39], found that the fecal microbiota of CDI patients displayed a distinct reduction in *Prevotella*, *Bacteroides*, and Bifidobacteria, and an increase in Enterobacteria, Clostridia, and Lactobacilli compared to other subject groups. Another study, conducted by Manges and colleagues, showed that a microbiota containing low levels of Bacteroidetes is significantly associated with CDI development [40]. We suggest that the increase in *Prevotella*, *Bacteroides*, and other species among the clade 1 CDI patients, which constituted the majority of CDI patients in this study, may be attributed to the high instability of the gut microbiota, a disequilibrium that could be induced by antibiotics. For example, in a study conducted by Barc and colleagues, treatment with amoxicillin/clavulanic acid increased the relative ratios of Bacteroidetes and the *Enterobacteriaceae* and caused a dramatic decrease in other species [41].

It is well-known that the gut microbiota is affected by antibiotic intake [5]. Antibiotic administration is a major cause of dysbiosis and has been linked to an increased risk of *C. difficile*-associated diarrhea. Nevertheless, gut microbial communities respond differently to different antibiotics. For example, in a study that tested the effect of antibiotics on the mouse microbiome, oral vancomycin did not decrease the abundance of mucosa-associated gut bacteria [42].

## 5. Conclusions

Although no direct associations between STs and disease severity were found, some associations indicate that different strains are associated with milder or more severe disease. For example, ST04 was characterized by a high resistance rate (93.75%) to moxifloxacin. As mentioned, a high resistance rate to this antibiotic could indicate an infection with a virulent strain or a therapeutic failure [8]. Regarding strains carrying the binary toxin gene, 45.5% of these strains were resistant to metronidazole and 55.5% were resistant to moxifloxacin. Furthermore, the only isolate that was resistant to vancomycin contained the binary toxin gene. When looking specifically at the NAP1 isolates, 100% were resistant to moxifloxacin and 66.66% were resistant to metronidazole. These findings suggest that strains producing binary toxin are more resistant to antibiotics.

To the best of our knowledge, no similar comprehensive study has yet been conducted in Israel. This study highlights the value of MLST databases for describing the different STs and clades of *C. difficile* isolates, and studying their unique characteristics. The resulting databases can enable clinicians to perform medical interventions based on the predicted response or disease severity associated with each strain, enabling personalized medicine.

## Figures and Tables

**Table 1:** Demographic characteristics of patients with *Clostridioides difficile* infection (CDI) and microbial characteristics of patient samples.

Patient Characteristic	N (%)
Gender	
Male	34 (48.6)
Female	36 (51.4)
Disease Severity	
Mild	45 (64.3)
Moderate	23 (32.8)
Severe	2 (2.9)
30-day mortality	
Alive	48 (68.6)
Died	22 (31.4)
Infection acquisition	
Nosocomial	47 (67.1)
Community	23 (32.9)
NAP1/027/B1	
Positive	3 (4.3)
Negative	67 (95.7)
Toxins *	
A	8 (11.4)
B	9 (12.9)
A + B	61 (87.1)

* Toxins results are presented according to the CerTest *Clostridium difficile* GDH + Toxin A + B combo card test kit.

**Table 2:** Prevalence of all *C. difficile* isolates resistant to various antimicrobial agents.

Antibiotic	Resistant Isolates n (%) *	Mean MIC_90_ (µg/mL)	MIC_50_ (µg/mL)
Metronidazole	12 (17.1)	40.6	0.5
Vancomycin	1 (1.4)	4.2	0.38
Moxifloxacin	29 (41.4)	13.7	2
Tigecycline	5 (7.1)	0.5	0.023

* Susceptibility was determined in accordance with the EUCAST recommendations, that determined epidemiological cut-offs (ECOFFs) [14].

**Table 3:** Associations between ST and epidemiological data.

Characteristic	ST04 (*n* = 16)	ST37 (*n* = 9)	ST104 (*n* = 6)	ST42 (*n* = 5)	ST02 (*n* = 5)	Other (*n* = 29)	*p*-Value
**Disease severity**							0.105
Moderate	4 # (25%)	5 (55.6%)	0 (0%)	0 # (0%)	3 (60%)	11 (37.9%)	
Mild	11 (68.8%)	4 (44.4%)	6 (100%)	4 (80%)	2 (40%)	18 (62.1%)	
**Gender**							0.979
Male	8 (50%)	5 (55.6%)	3 (50%)	2 (40%)	3 (60%)	13 (44.8%)	
Female	8 (50%)	4 (44.4%)	3 (50%)	3 (60%)	2 (40%)	16 (55.2%)	
**Disease acquisition**							0.326
Nosocomial	13 (81.3%)	6 (66.7%)	3 (50%)	4 (80%)	2 (40%)	20 (69%)	
Community	3 (18.7%)	3 (33.3%)	3 (50%)	1 (20%)	3 (60%)	9 (31%)	
**30-day mortality**							0.641
Alive	10 (62.5%)	6 (66.7%)	6 (100%)	3 (60%)	3 (60%)	20 (69%)	
Died	6 (37.5%)	3 (33.3%)	0 (0%)	2 (40%)	2 (40%)	9 (31%)	
**Number of Classes**							0.761
0	0 (0%) 10	0 (0%)	0 (0%)	0 (0%)	0 (0%)	1 (3.4%) #	
1	(62.5%)	2 (22.2%)	2 (33.3%)	2 (40%)	3 (60%)	15 (51.7%)	
2	1 (6.2%)	3 (33.3%)	1 (16.7%)	2 (40%)	2 (40%)	2 (6.9%)	
3	3 (18.8%)	2 (22.2%)	1 (16.7%)	0 (0%)	0 (0%)	9 (31%)	
4	1 (6.2%)	0 (0%)	1 (16.7%)	0 (0%)	0 (0%)	1 (3.4%)	

# Patients with severe illness were excluded from analysis due to their small number (2).

**Table 4:** Associations between ST and bacterial characteristics.

Characteristics	ST04 (*n* = 16)	ST37 (*n* = 9)	ST104 (*n* = 6)	ST42 (*n* = 5)	ST02 (*n* = 5)	Other (*n* = 29)	*p*-Value
**Toxins #**							0.906
A	1 (6.3%)	1 (11.1%)	0 (0%)	1 (20%)	0 (0%)	5 (17.2%)	
B	7 (43.7%)	3 (33.3%)	2 (33.3%)	1 (20%)	1 (20%)	9 (31%)	
A + B	8 (50%)	5 (55.6%)	4 (66.7%)	3 (60%)	4 (80%)	15 (51.8%)	
**Binary toxin**							0.614
Negative	14 (87.5%)	7 (77.8%)	6 (100%)	5 (100%)	5 (100%)	24 (82.7%)	
Positive	2 (12.5%)	2 (22.2%)	0 (0%)	0 (0%)	0 (0%)	5 (17.3%)	
**Metronidazole**							0.550
S	13 (81.3%)	6 (66.7%)	6 (100%)	5 (100%)	4 (80%)	24 (82.7%)	
R	3 (18.7%)	3 (33.3%)	0 (0%)	0 (0%)	1 (20%)	5 (17.3%)	
**Moxifloxacin**							0.001 *
S	1 (6.3%)	5 (55.6%)	6 (100%)	5 (100%)	2 (40%)	22 (75.8%)	
R	15 (93.7%)	4 (44.4%)	0 (0%)	0 (0%)	3 (60%)	7 (24.2%)	
**Tigecycline**							0.626
S	16 (100%)	8 (88.9%)	5 (83.3%)	5 (100%)	5 (100%)	26 (89.7%)	
R	0 (0%)	1 (11.1%)	1 (16.7%)	0 (0%)	0 (0%)	3 (10.3%)	
**Vancomycin**							0.230
S	16 (100%)	8 (88.9%)	6 (100%)	5 (100%)	5 (100%)	29 (100%)	
R	0 (0%)	1 (11.1%)	0 (0%)	0 (0%)	0 (0%)	0 (0%)	

* Bold values indicate statistical significance. # Toxins results are presented according to the CerTest *Clostridium difficile* GDH + Toxin A + B combo card test kit.

**Table 5:** Association between binary toxin gene presence and bacterial characteristics.

Characteristic	Binary-Negative Isolates (*n* = 61) N (%)	Binary-Positive Isolates (*n* = 9) N (%)	*p*-Value
**NAP1**			0.001 *
Positive	61 (100)	6 (66.7)	
Negative	0 (0)	3 (33.3)	
**Toxins**			0.449
A	8 (13.1)	0 (0)	
B	19 (31.1)	4 (44.5)	
A + B	34 (55.7)	5 (55.5)	
**Metronidazole**			**0.020**
S	53 (86.9)	5 (55.5)	
R	8 (13.1)	4 (45.5)	
**Moxifloxacin**			**0.009**
S	37 (60.7)	4 (44.5)	
R	24 (39.3)	5 (55.5)	
**Tigecycline**			0.357
S	57 (93.4)	8 (88.9)	
R	4 (6.6)	1 (11.1)	
**Vancomycin**			0.620
S	61 (100)	8 (88.9)	
R	0 (0)	1 (11.1)	

* Bold values indicate statistical significance.

## Supplementary Materials

The following are available online at https://www.mdpi.com/2077-0383/9/12/4099/s1, Table S1: Association between binary toxin gene presence and demographics data.
