# Supplementary material for: Characterization of Clostridioides difficile Strains, the Disease Severity, and the Microbial Changes They Induce
Source: J Clin Med. 2020 Dec 18;9(12):4099. doi: 10.3390/jcm9124099 (PMC7766075; doi:10.3390/jcm9124099)
Supplement: Supplementary file 1 [file jcm-09-04099-s001.pdf]

Table S1. Association between binary toxin gene presence and demographics data.

**Characteristic**	**Non-binary (*n* = 61)**	**Binary (*n* = 9)**	**p-value**
**Age**			
<65	10 (16.4%)	0 (0%)	
75–65	23 (37.7%)	5 (55.5%)	0.349
75+	28 (45.9%)	4 (44.5%)	
**Gender**			
Male	29 (47.5%)	5 (55.5%)	0.653
Female	32 (52.5%)	4 (44.5%)	
**Nosocomial/Community-acquired**			
Nosocomial	42 (68.9%)	6 (66.7%)	0.895
Community	19 (31.1%)	3 (33.3%)	
**Death in hospital**			
Alive	43 (70.5%)	5 (55.5%)	0.368
Died	18 (29.5%)	4 (44.5%)	
